# Physical activity profiles in Parkinson’s disease

**DOI:** 10.1186/s12883-021-02101-2

**Published:** 2021-02-13

**Authors:** Philip von Rosen, Maria Hagströmer, Erika Franzén, Breiffni Leavy

**Affiliations:** 1 Department of Neurobiology, Care Sciences and Society, Division of Physiotherapy, Karolinska Institutet, Alfred Nobels Allé 23, 141 83 Huddinge, Sweden; 2 Academic Primary Care Center, Region Stockholm, Stockholm, Sweden; 3 Department of Health Promoting Science, Sophiahemmet University, Stockholm, Sweden; 4 Karolinska University Hospital, Medical unit Occupational Therapy & Physiotherapy, Theme Women’s Health and Allied Health Professionals, Stockholm, Sweden; 5 Stockholms Sjukhem Foundation, Reseach and Development Department, Stockholm, Sweden

**Keywords:** Physical activity, Sedentary behaviour, Accelerometry, Parkinson’s disease

## Abstract

**Background:**

Identifying physical activity (PA) profiles of people with Parkinson’s Disease (PD) could provide clinically meaningful knowledge concerning how to tailor PA interventions. Our objectives were therefore to i) identify distinct PA profiles in people with PD based on accelerometer data, ii) explore differences between the profiles regarding personal characteristics and physical function.

**Methods:**

Accelerometer data from 301 participants (43% women, mean age: 71 years) was analysed using latent profile analyses of 15 derived PA variables. Physical function measurements included balance performance, comfortable gait speed and single and dual-task functional mobility.

**Results:**

Three distinct profiles were identified; “*Sedentary*” (*N* = 68), “*Light Movers*” (*N* = 115), “*Steady Movers*” (*N* = 118). “*Sedentary*” included people with PD with high absolute and relative time spent in Sedentary behaviour (SB), little time light intensity physical activity (LIPA) and negligible moderate-to-vigorous physical activity (MVPA). “*Light Movers*” were people with PD with values close to the mean for all activity variables. “*Steady Movers*” spent less time in SB during midday, and more time in LIPA and MVPA throughout the day, compared to the other profiles. “*Sedentary*” people had poorer balance (*P* = 0.006), poorer functional mobility (*P* = 0.027) and were more likely to have fallen previously (*P* = 0.027), compared to “*Light Movers*. The Timed Up and Go test, an easily performed clinical test of functional mobility, was the only test that could distinguish between all three profiles.

**Conclusion:**

Distinct PA profiles, with clear differences in how the time awake is spent exist among people with mild-moderate PD.

## Background

People with Parkinson’s disease (PD) have much to gain from engaging in a physically active lifestyle, in terms of managing and potentially modifying the rate of symptom progression [1, 2]. The benefits of physical activity (PA) at mild-moderate disease stages are strongly supported in the literature, with level one evidence for improvements in aspects of physical performance such as, gait, muscle strength and cardiovascular endurance in the short [1, 3–7], and in the long-term [8]. Additionally, it appears that exercise, particular when aerobic in nature [9, 10], can improve global cognition [11, 12] and reduce depression [13] – neuropsychiatric features which become increasingly prevalent with disease progression [14, 15] – although larger-scale studies are required [1].

PA is a multidimensional behaviour that can be described using a variation of measures such as, absolute or relative time spent in different intensities of sedentary behaviour (SB), light intensity physical activity (LIPA) and moderate-to-vigorous physical activity (MVPA), by total time in bouts or through variation over a day. Despite the proven benefits of PA on motor and non-motor symptoms, people with PD are generally less physically active than people of similar age without the disease. Community-dwellers who are independently ambulatory take approximately 5000 steps/day [16–18], even those who are newly diagnosed [19] and prior to the commencement of anti-Parkinson medications [20]. When viewed in relation to the approximated 7000 steps/day required of older adults in order to meet health recommendations [21], the activity patterns of people with PD reflect sedentary lifestyles [22]. A single measure, such as steps/day however, inadequately depicts PA as it fails to indicate whether activity occurs at sufficient intensity or duration to benefit health. A major challenge for people with PD is the achievement of sustained bouts of MVPA [17–19], a pattern even apparent among those with higher step counts [23]. On average, people with PD appear to spend 75% of their waking time in SB, and between 2 and 6% of their total time in MVPA [16, 17, 24].

Not only are people with PD at risk of physical inactivity early in the disease, it is likely that decreasing PA levels will go unnoticed in the clinical context, as activity levels appear to decline prior to deteriorations in clinical tests of motor impairment or gait speed [25, 26]. Additionally, giving PA advice based on Hoehn & Yahr (H&Y) disease stage is unlikely to be effective as disease severity does not have a large association with activity levels [19]. Although widely acknowledged that people at similar disease stages can have different PA behaviors, this heterogeneity of PA patterns in PD is poorly reflected in the litterature. Previous studies investigating PA patterns in PD are often based on small samples and have reported results for entire samples.

Latent profile analysis is a statistical method that can be used to identify heterogeneous groups of people with PD, based on response patterns for multiple objective measures of PA [27]. This method enables the grouping of people into mutually exclusive PA profiles, as has been performed in PA research among people living with other forms of chronic progressive disease [28, 29]. Identifying PA profiles among people with PD could provide clinically meaningful knowledge concerning PA patterns which in turn could enable clinicians to tailor PA interventions. The primary aim of this study was therefore to identify distinct PA profiles among people with PD based on accelerometer measured PA. In order to validate the PA profiles, the secondary aim was to explore differences between the profiles regarding personal characteristics and physical function.

## Methods

### Study population and design

This study used baseline data from the combined cohorts of three stages of the BETA-PD project. Participants were recruited through advertisement in local papers, contact with patient associations, as well as from waiting lists at four clinical sites specialising in neurological rehabilitation. Participants were included if they were; diagnosed with idiopathic PD by a neurologist; at mild-moderate disease stages (H&Y stages 2 and 3); stable in their anti-PD medication and were ambulatory. Participants were excluded if they had co-existing neurological or orthopedic conditions affecting gait or balance or were cognitively impaired (Mini Mental State Examination score < 24 points or Montreal Cognitive Assessment score ≤ 21 points). Data collection was approved by the Regional Ethical Review Board in Stockholm. Trials were registered at clinicaltrials.gov with clinical trial numbers NCT10417598, NCT02727478 and NCT03213873.

### Data collection

Demographic and anthropometric data on age, body mass index (BMI), falls within 12 months were collected from a baseline questionnaire.

#### Physical activity

The ActiGraph accelerometer model GT3X+ (ActiGraph, Pensacola, FL, US) was used to capture time in different behaviours, measuring time-varying acceleration in the vertical axis expressed as counts. The participants were instructed to wear the accelerometer on the hip for seven consecutive days. The device was set to sampling counts per 1-min epochs. Non-wear time was defined as periods of at least 60 consecutive minutes of zero counts, allowing for 2 min of counts between zero and 100. Data from participants with at least one valid day, including 10 h or more of wear time, were included. Epochs were classified into intensity levels using validated cut-points chosen in accordance with validated cut-points for older adults: SB (< 100 cpm), LIPA (100–1040 cpm) and MVPA (≥ 1041 cpm) [30]. Performed time in ≥30-min bouts of SB and performed time in ≥10-min bouts of MVPA, was calculated. Number of SB bouts (lasting ≥30-min) and MVPA bouts (lasting ≥10-min) were estimated. The software ActiLife 6 (ActiGraph, Pensacola, FL, US) was used to extract and process the accelerometer data.

#### Physical function

All tests were performed by qualified physical therapists during participants’ “ON” medication cycle – 1 to 2 h after taking their anti-Parkinson medication. Testing protocol commenced with an interview and was followed by performance tests of gait and balance. Balance performance was measured using the Mini-Balance Evaluation Systems Test (Mini-BESTest), which is recommended for use in PD and assesses 4 balance subdomains with a maximum score of 28 points [31]. Comfortable gait speed was measured using an electronic walkway system (GAITRite®, CIR Systems Inc., PA) and the 10 Meter Walk test. Single- and dual-task functional mobility was captured using the Timed-Up and Go (TUG) test and the Cognitive TUG test (TUG COG). The TUG test is a clinical test which assesses the sequential performance of rising from a chair, walking 3 m, turning and walking back to the chair, is reliable for PD, and can detect differences in performance [32].

### Data analysis

#### Physical activity variables

Fifteen different PA variables capturing a wide range of characteristics and based on previous literature [28, 33], were derived from accelerometer data. These included time spent in different intensities (SB, LIPA, MVPA), relative time spent in one behaviour in relation to two remaining behaviours (e.g. Relative time MVPA), total time of sedentary/MVPA bouts, number of sedentary/MVPA bouts, the change of time in SB and MVPA between evening-afternoon (Change SB/MVPA evening) and afternoon-morning (Change SB/MVPA afternoon), and total counts. The change of time spent in SB/MVPA was calculated as the difference in time between evening/afternoon and afternoon/morning of SB and MVPA, respectively, where morning was defined as 6 am – 12 am, afternoon as 12 am – 6 pm and evening as 6 pm – 12 pm. The relative time in one behaviour was calculated as isometric log-ratio coordinates [34]. Three variables were derived representing the time in one behaviour (eg. MVPA) relative to the average of the two other behaviours (eg. SB, LIPA). All 15 activity variables were calculated across all valid days and transformed into z-scores. Spearman correlations between the 15 variables were used to identify multicollinearity. This resulted in the exclusion of total time of MVPA bouts, number of sedentary/MVPA bouts, total counts and the relative time of MVPA, from the analysis.

#### Latent profile analysis

Several consecutive latent models with two to six profile solutions were performed using latent profile analysis from package “tidyLPA” in R. The best model according to the following fit statistics was chosen: 1) Akaike Information Criterion (AIC) and the Bayesian Information Criterion (BIC), 2) Entropy values, 3) Probability of profile membership, 4) Smallest group including more than 10% of all participants, 5) meaningfulness of profile membership. Based on the fit statistics a 3-profile solution was chosen. Results of the different consecutive latent profile models with 2 to 6-profile solutions are presented in Table 1.

**Table 1:** Fit indices of the 2 to 6-profile latent class models

Profiles	AIC	BIC	Lowest mean value of posterior probability in each profile	Entropy	Smallest group size
2	7649	7801	0.97	0.89	0.42
3	7179	7409	0.96	0.92	0.23
4	6981	7289	0.94	0.91	0.23
5	6784	7170	0.93	0.92	0.16
6	6683	7146	0.94	0.94	0.10

*AIC* Akaike Information Criterion, *BIC* Bayesian Information Criterion

A multinomial regression analysis was conducted to model profile membership. Possible independent variables included age, BMI, falls within 12 months (yes/no), gait speed, Mini-BESTest, sex, time in years since PD diagnosis, TUG test and TUG COG and walking aid use (yes/no). Missing data accounted for less than 17% of all cases. A total of five datasets were imputed based on a chained equation algorithm. The pooled dataset was then compared to complete case analysis which showed an overall small change in beta coefficients (< 5%) for all variables, except use of walking aid (< 10.5%). This was not considered to have any appreciable effects on the final model and therefore it was chosen to report the results of the multinomial regression analysis using the pooled dataset.

All independent variables associated with the dependent variable at *P* < 0.20, in univariate regression analyses, were included in a backward multinomial regression analysis. Independent variables *P* > 0.10 were then removed. Finally, all independent variables excluded in the univariate regression analyses were included one by one in the multinomial regression analysis, and kept only if *P* ≤ 0.10. Hosmer-Lemeshow test was used to assess goodness of fit. The analyses were conducted using the R statistical system version 3.5.2 (R Core Team 2018).

## Results

**Fig. 1:**
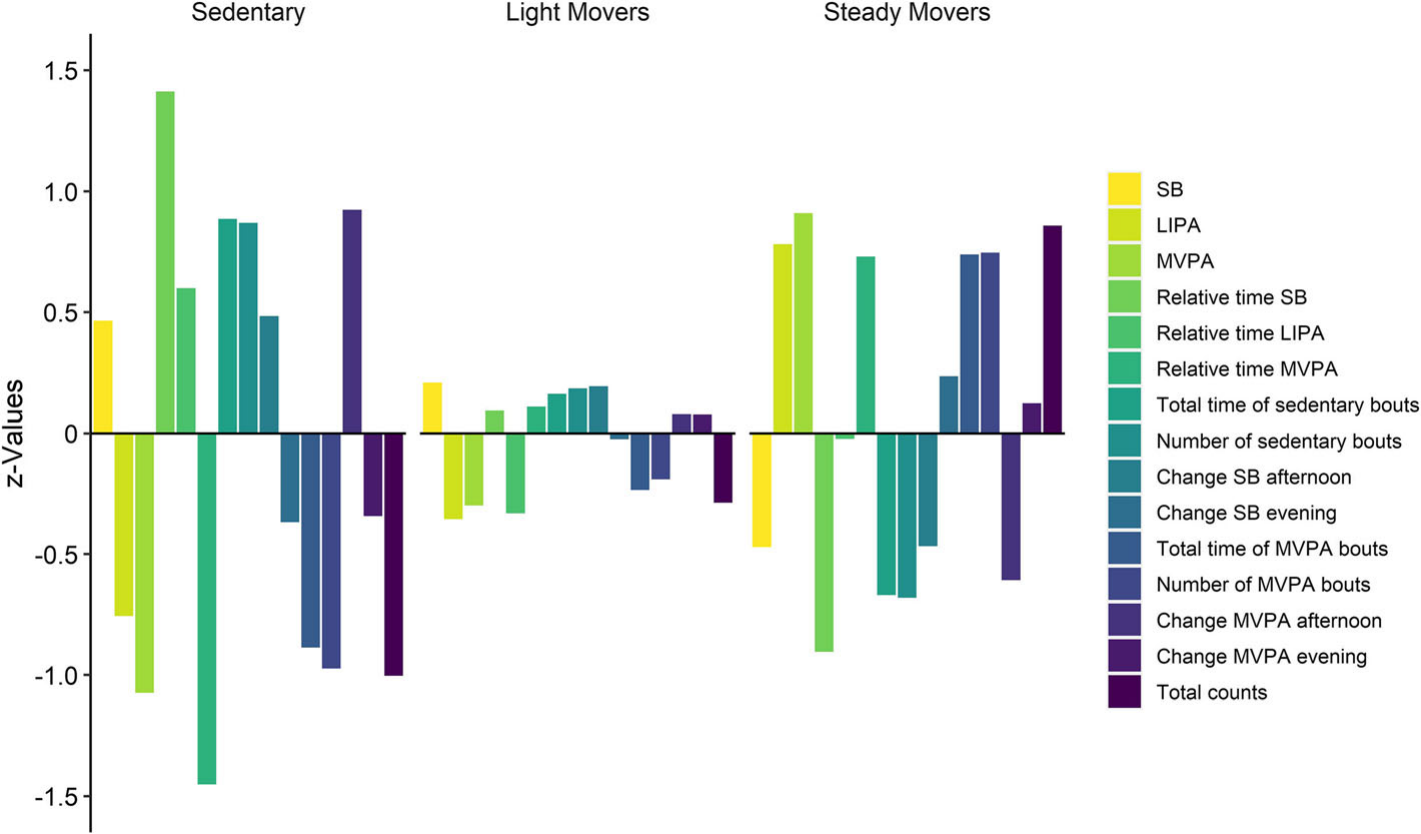
Z-values for different activity variables by physical activity profiles, with z equal to 0 as the mean value of the complete population. LIPA, light intensity physical activity; MVPA, moderate-to-vigorous physical activity; SB, sedentary behavior

**Fig. 2:**
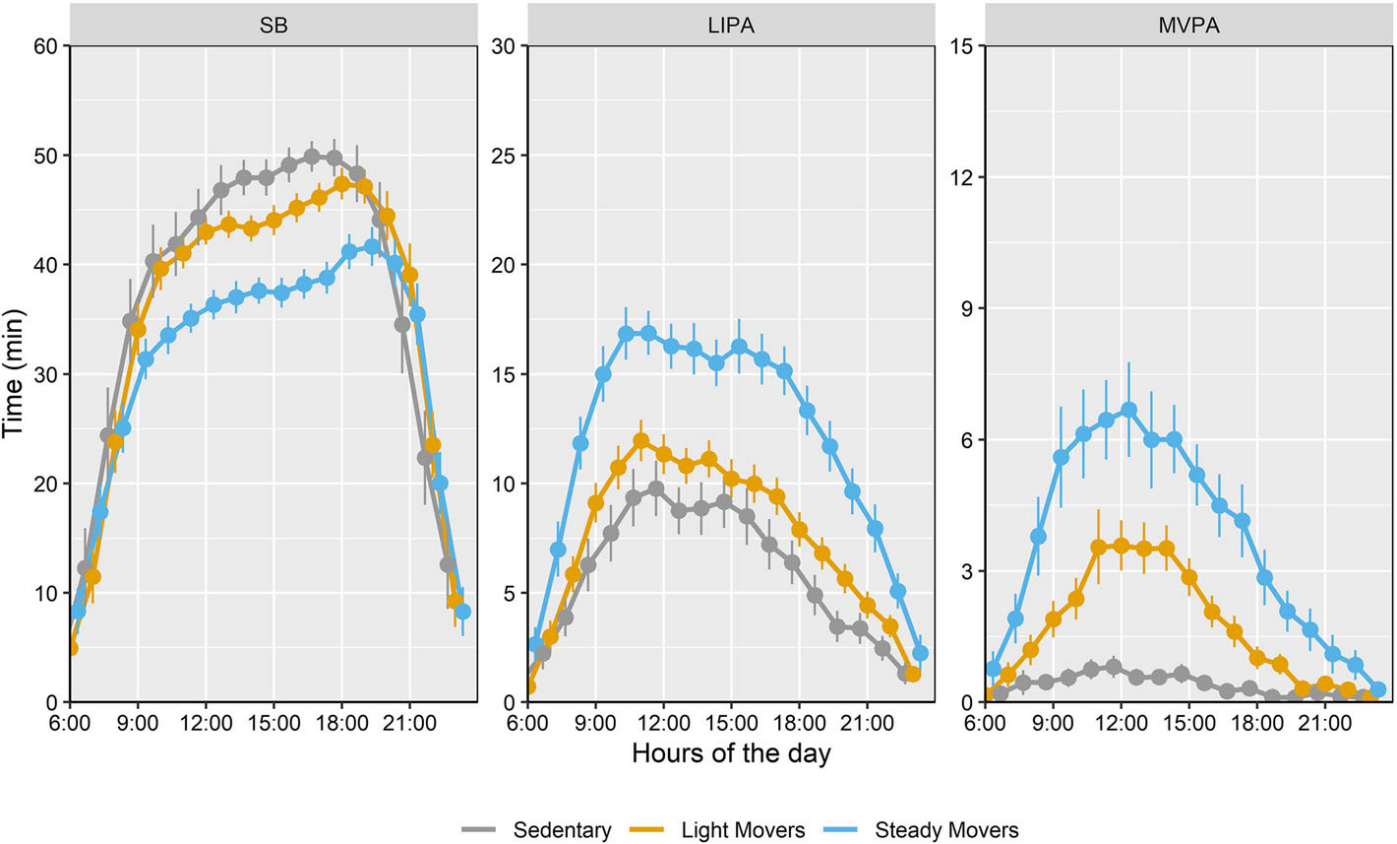
Mean time spent in physical activity and sedentary behaviour (SB) by hour (95% confidence intervals shown as error bars) for different physical activity profiles. LIPA, light intensity physical activity; MVPA, Moderate-to-vigorous physical activity

Descriptive data for the total population (*N* = 301) are presented in Tables 2 & 3. Data analysis resulted in three distinct PA profiles. Profile 1 (*N* = 68, 23%) was named “*Sedentary*” to reflect people with PD with high absolute and relative time spent in SB and little time spent in LIPA and negligible MVPA (Fig. 1). These people spent more time in SB during the afternoon and consistently very low time in MVPA across most parts of the day, compared to the other two profiles (Fig. 2). Profile 2 (*N* = 115, 38%) named “*Light Movers*”, was characterized by people with PD with values close to the mean (< 0.5 z-score) for all activity variables. These people spent more time in LIPA and MVPA for most hours of the day compared to the “*Sedentary*” profile. Profile 3 (*N* = 118, 39%) was named “*Steady Movers*” since these people with PD spent less time in SB during the middle of the day, and more time in LIPA and MVPA for most parts of the day, compared to both “*Sedentary*” and “*Light Movers*” profiles.

**Table 2:** Descriptive data for the study population and by profile membership

Characteristic	Total Population (*n* = 301)	Sedentary (*n* = 68)	Light Movers (*n* = 115)	Steady Movers (*n* = 118)
Age, mean (SD)	71.4 (6.4)	73.5 (5.1)	71.7 (6.3)	69.8 (6.7)
Sex (female), n (%)	130 (43)	25 (37)	49 (43)	56 (48)
BMI, mean (SD)	25.0 (3.5)	25.3 (4.0)	25.5 (3.2)	24.4 (3.6)
Fall within 12 months, n (%)	137 (48)	35 (52)	58 (55)	44 (40)
Gait speed m/s, mean (SD)	1.1 (0.3)	1.0 (0.2)	1.1 (0.3)	1.1 (0.3)
Hoehn and Yahr Scale, n (%)				
Stage 2	162 (54)	21 (31)	59 (52)	82 (70)
Stage 3	136 (46)	46 (69)	55 (48)	35 (30)
MiniBESTest, mean (SD)	20.3 (3.7)	17.9 (3.7)	20.2 (3.5)	21.7 (3.0)
Timed Up & Go Test, mean (SD)	10.8 (2.7)	12.7 (3.4)	11.0 (2.5)	9.6 (1.7)
Cognitive Timed Up & Go, mean (SD)	17.5 (12.2)	18.8 (10.0)	19.7 (16.2)	14.6 (7.3)
Use of walking aid, n (%)	88 (31)	32 (48)	38 (36)	18 (16)
Years since PD diagnosis, mean (SD)	6.6 (5.0)	7.4 (6.1)	5.6 (3.9)	7.0 (5.2)

*SD* Standard deviation, *BMI* Body mass index, *m/s* Meters per second, *PD* Parkinson’s disease

**Table 3:** Descriptive data over activity variables with mean values (SD) for the study population and by profile membership

	Total Population (*n* = 301)	Sedentary (*n* = 68)	Light Movers (*n* = 115)	Steady Movers (*n* = 118)
SB min/day	605.6 (97.7)	650.9 (101.6)	625.9 (90.8)	559.6 (82.2)
LIPA min/day	158.1 (71.8)	103.8 (48.0)	132.6 (45.4)	214.2 (66.1)
MVPA min/day	38.6 (29.6)	6.8 (3.9)	29.8 (11.4)	65.6 (26.8)
Relative time SB	1.9 (0.6)	2.7 (0.4)	1.9 (0.2)	1.3 (0.3)
Relative time LIPA	0.1 (0.4)	0.4 (0.5)	−0.05 (0.4)	0.1 (0.4)
Relative time MVPA	−2.0 (0.8)	−3.1 (0.6)	−1.9 (0.4)	−1.4 (0.4)
Total time of sedentary bouts min/day	273.1 (124.5)	383.5 (119.3)	293.3 (108.0)	189.9 (75.9)
Number of sedentary bouts	9.2 (3.6)	12.4 (3.2)	9.9 (3.0)	6.7 (2.6)
Change SB afternoon	102.0 (55.8)	128.9 (63.2)	112.8 (48.9)	75.9 (46.3)
Change SB evening	− 53.6 (59.6)	−75.5 (65.6)	−55.0 (57.6)	−39.6 (54.0)
Total time of MVPA bouts min/day	19.4 (21.0)	0.7 (1.6)	14.5 (10.7)	34.9 (23.5)
Number of MVPA bouts	1.0 (1.0)	0.1 (0.1)	0.8 (0.5)	1.8 (1.1)
Change MVPA afternoon	6.2 (15.8)	−2.3 (2.5)	−14.1 (7.6)	−23.7 (16.5)
Change MVPA evening	−15.2 (14.0)	0.8 (3.0)	7.4 (11.7)	8.2 (21.9)
Total counts	2868.9 (1784.9)	1077.2 (560.5)	2357.3 (1062.0)	4399.9 (1550.1)

*SB* Sedentary behaviour, *LIPA* Light intensity physical activity, *MVPA* Moderate-to-vigorous physical activity

### Profile characteristics

People belonging to the “*Sedentary*” profile had greater odds of having poorer balance (*P* = 0.006), poorer functional mobility (*P* = 0.027) and were more likely to have fallen in the previous year (*P* = 0.027), compared with the “*Light Movers*” (Table 4). Those belonging to the “*Steady Movers*” were more likely to have better single (*P* = 0.008) and dual-task functional mobility (*P* = 0.036) and a lower BMI (*P* = 0.029) than “*Light Movers*”. Additionally, this sub-group were more likely to be at H & Y stage 2 (*P* = 0.034), yet have lived with the diagnosis for a greater number of years (*P* = 0.023) compared to “*Light Movers*”.

**Table 4:** Multinomial regression analysis for profile membership

	Sedentary vs. Light Movers^a^			Steady Movers vs. Light Movers^a b^		
Model	OR (95% CI)	Wald	*p*-value	OR (95% CI)	Wald	*p*-value
BMI	0.97 (0.89–1.06)	0.44	0.505	0.91 (0.83–0.99)	4.78	0.029*
Fall within 12 months (reference no fall)	2.28 (1.10–4.72)	4.93	0.027*	1.76 (0.95–3.27)	3.18	0.075
Hoehn and Yahr Scale (reference stage 3)	0.64 (0.31–1.33)	1.43	0.231	2.02 (1.06–3.86)	4.52	0.034*
Mini-BESTest	0.84 (0.75–0.95)	7.51	0.006*	1.00 (0.89–1.11)	0.006	0.936
Timed Up & Go Test	1.18 (1.02–1.37)	4.90	0.027*	0.80 (0.68–0.95)	6.99	0.008*
Cognitive Timed Up & Go test	0.96 (0.92–1.00)	3.21	0.074	0.96 (0.93–0.998)	4.40	0.036*
Years since PD diagnosis	1.07 (1.00–1.15)	3.80	0.052	1.08 (1.01–1.15)	5.16	0.023*

*CI* Confidence interval, *OR* Odds ratio, *PD* Parkinson’s disease; ^a^ Intercept b = 0.60; ^b^ Intercept b = 4.35

* Significant at *p* ≤ 0.05

## Discussion

To the best of our knowledge, this is the first study to identify three distinct and meaningfully interpretable PA profiles in PD, derived from objectively measured data using latent profile analysis. The “Sedentary” profile spent a high proportion of their time in SB during the afternoon and a consistent minimal amount of time in MVPA across the entire day. Although “*Light Movers*” showed similar hourly patterns of time spent in SB and LIPA as “*Sedentary*” people, they spent more time in LIPA and MVPA compared to them. “*Steady Movers*” spent more time in LIPA and MVPA for most of the day, compared to the other profiles. The TUG test, a quick and commonly used clinical test of functional mobility, was the only test that could distinguish between all three profiles, with a difference of approximately 1.5 s between the nearest profile.

Our latent profile analysis highlights the extent of physical inactivity existing even among groups of people with PD who actively seek rehabilitation. “*Sedentary”* people achieved only a few minutes of time in MVPA and spent 99% of their awake time in SB and/or LIPA, which is comparative to reports from self-identified sedentary PD samples [35]. Poor balance capacity, poor functional mobility and having a history of falls predicted belonging to the “*Sedentary*” profile. This finding is supported in the literature, as H&Y score [19, 24] and gait speed [19] have been reported as predictors of poor PA levels and having fallen is associated with SB in people with PD [36].

Although this group spent time in LIPA in the morning, this activity drops steadily from midday onwards, explaining a total activity count that is 75% lower than “*Steady Movers*”. It is previously reported that prolonged bouts of SB appear characteristic of more advanced PD compared to controls [37] and these uninterrupted bouts also appear more strongly associated with quality of life in this group [38]. A feasible starting point for PA promotion for this group, could be to break up bouts of prolonged sitting in the afternoon and evening time. The gradual replacement of 30 min of SB with LIPA, would in turn increase their activity levels to those of “*Light Movers”*. Substituting 30 min of SB is estimated to reduce all-cause and cardiovascular-related mortality [39, 40], and substitution with non-exercise related activity, such as household chores or gardening may benefit sedentary individuals especially [41]. Additionally, physically inactive people with PD often perceive many barriers to and have low outcome expectations for exercise [42, 43], and may therefore be more receptive to messages focusing on breaking sitting bouts with non-exercise recreational activities. Although, “*Sedentary*” people accounted for almost every fourth person in this study, in consideration that our sample consisted of people who actively sought rehabilitation, it is likely that a larger proportion of the PD population belong to this category.

“*Light Movers*” achieved approximately 30 min of MVPA/day, which is in line with recommended health guidelines for PA [44]. Nonetheless, this group could achieve greater health benefits if a higher proportion of time spent in SB is replaced with time in MVPA. After 3 PM, a reduction of time spent in LIPA and LIPA/MVPA was observed among “*Sedentary*” and “*Light Movers*” respectively. A previous study of hourly PA patterns, although involving a small PD sample, showed a second distinct evening time peak in PA [45]. A realistic aim for “*Light Movers*” could be to achieve this second peak in time spent in LIPA or MVPA after 3 PM, by engaging in activities like brisk walking, household chores or gardening, a change that would also align their PA closer to levels of “*Steady Movers*”.

“*Steady Movers*” consistently maintained 5–6 min of MVPA every hour between the morning and the afternoon, which accumulated to a daily 60 min, spent in MVPA. It has been previously reported that, declines in MVPA account for the largest decline in walking behaviour in PD over the course on 1 year [25], highlighting the importance of encouraging these individuals to maintain their PA behaviour. Although the majority of “*Steady Movers*” were at mild disease stages, people at moderate stages accounted for one third of this profile. This group appears therefore to include people with PD who have succeeded in maintaining levels of MVPA despite disease progression.

The strengths of this study include the availability of objectively measured PA data from a large cohort of people with PD, and the use latent profile analysis based on a comprehensive range of activity variables. Additionally, PA profiles were validated using data on both personal characteristics and physical function assessed through reliable and clinically relevant tests. Several limitations should also be acknowledged. Our findings are limited to people with PD who actively seek rehabilitation with mild-moderate disease stages and without cognitive impairment or co-existing neurological or orthopedic conditions affecting gait and balance. PA data is cross-sectional in nature and it is therefore not possible to explore if these behaviours were constant over time.

In addition, accelerometers do not measure all kinds of PA constructs (e.g., bicycling, strength training or swimming). Multiple PA variables were used in latent profile analysis, however there are other potential measures of PA (e.g. variation between days, different lengths of bouts) that could have been derived from accelerometer data to describe the profiles. It is also possible that non-motor symptoms such as, fear of falling, depression and fatigue, as well as cardiovascular functioning, not accounted for in this study, could be associated with the low levels of MVPA observed in the “Sedentary” profile [18, 20, 46].

## Conclusion

Based on PA variables and time spent in PA and SB by hour across the day, three distinct PA profiles were detected in people with mild-moderate PD, which had clear differences in how time awake was spent. The “Sedentary” profile spent 99% of their awake time in SB or LIPA. People belonging to this profile had poorer balance, poorer functional mobility and were more likely to have fallen in the previous year, compared with the “*Light Movers*”. The TUG test – an easily administered clinical test of functional mobility – was the only test that could distinguish between all three profiles. Practical recommendations for promoting a physically active lifestyle for each profile are presented in Table 5. Our findings provide evidence for diversity of PA behavior among those with PD who actively seek rehabilitation and provide important insights for developing and tailoring PA interventions among this diverse group.

**Table 5:** Practical recommendations for promoting a physically active lifestyle by profile membership

Profile	Recommendation	Rationale
Sedentary	Break up bouts of prolonged sitting by engaging in activities like walking, light household chores or activities like dance, etc.	To replace time in SB with time spent in LIPA or, if possible, time in MVPA.
Light Movers	Engage in activities like brisk walking, household chores or gardening at least twice a day.	To achieve a second peak of time spent in LIPA or MVPA.
Steady Movers	Continue to engage in activities such as brisk walking, gardening, cycling, etc.	To maintain time spent in MVPA.

*SB* Sedentary behaviour, *LIPA* Light intensity physical activity, *MVPA* Moderate-to-vigorous physical activity

## Data Availability

The datasets generated during and/or analysed during the current study are not publicly available due to Swedish and EU personal data legislation but are available from the corresponding author on reasonable request. Any sharing of data will be regulated via a data transfer and user agreement with the recipient.

## Abbreviations

People with PD: People with Parkinson’s disease
PA: Physical activity
SB: Sedentary behaviour
LIPA: Light intensity physical activity
MVPA: Moderate-to-vigorous physical activity (MVPA)
H&Y: Hoehn & Yahr
BMI: Body mass index
Mini-BESTest: Mini-Balance Evaluation Systems Test
TUG: Timed-Up and Go test
TUG COG: Cognitive Timed-Up and Go test
